# Convolutional Neural Network and Bidirectional Long Short-Term Memory-Based Method for Predicting Drug–Disease Associations

**DOI:** 10.3390/cells8070705

**Published:** 2019-07-11

**Authors:** Ping Xuan, Yilin Ye, Tiangang Zhang, Lianfeng Zhao, Chang Sun

**Affiliations:** 1 School of Computer Science and Technology, Heilongjiang University, Harbin 150080, China; 2 School of Mathematical Science, Heilongjiang University, Harbin 150080, China

**Keywords:** drug repositioning, convolutional neural network, drug research and development, bidirectional long short-term memory, attention mechanism at path level

## Abstract

Identifying novel indications for approved drugs can accelerate drug development and reduce research costs. Most previous studies used shallow models for prioritizing the potential drug-related diseases and failed to deeply integrate the paths between drugs and diseases which may contain additional association information. A deep-learning-based method for predicting drug–disease associations by integrating useful information is needed. We proposed a novel method based on a convolutional neural network (CNN) and bidirectional long short-term memory (BiLSTM)—CBPred—for predicting drug-related diseases. Our method deeply integrates similarities and associations between drugs and diseases, and paths among drug-disease pairs. The CNN-based framework focuses on learning the original representation of a drug-disease pair from their similarities and associations. As the drug-disease association possibility also depends on the multiple paths between them, the BiLSTM-based framework mainly learns the path representation of the drug-disease pair. In addition, considering that different paths have discriminate contributions to the association prediction, an attention mechanism at path level is constructed. Our method, CBPred, showed better performance and retrieved more real associations in the front of the results, which is more important for biologists. Case studies further confirmed that CBPred can discover potential drug-disease associations.

## 1. Introduction

The research and development (R&D) stage of producing a novel drug is a time-consuming, complex, and costly process that normally lasts for more than ten years and costs approximately 1 billion dollars [1,2,3,4]. Simultaneously, there is a large gap between the high investment in R&D and the number of new drugs finally approved [5,6,7]. Because approved drugs have undergone the necessary clinical trials, their safety has been evaluated, identifying new indications for these drugs, (i.e., drug repositioning), which can effectively reduce the time and costs for drug-related R&D [5,8,9].

Network-based approaches have been widely used to study biological and medical associations [10,11]. Computational prediction of the associations between drugs and diseases can identify candidates for further wet-lab validation [12,13]. Several methods are used to predict and prioritize drug-associated diseases, which can generally be divided into two categories. Methods in the first category capture network topology information using a diffusion algorithm and then provide association scores for candidate diseases [14,15,16,17]. Wang et al. [16] identified candidate diseases using an iterative update algorithm based on the guilt-by-association principle. Luo et al. [15] established a drug network and disease network and calculated association scores by random walk of the two networks. Liu et al. [14] integrated the two networks as a drug–disease network and applied a random walk method to the network. These methods inferred candidates with edges weighted by similarities and associations among nodes in the network. However, a major limitation to these approaches is that they only consider the topological information of the network while ignoring original information at the nodes. 

Methods in the second category mainly integrate the heterogeneous similarities of drugs or diseases through matrix factorization and projection [1,18]. A method developed by Liang et al. [1] works by minimizing the loss of the prediction matrix from the original association matrix from various perspectives. Zhang et al. [18] considered the biological background using the similarities of drugs and diseases as a constraint for low-dimensional matrices during prediction. However, in these methods, low-frequency effective information may be missed during the projection process. Additionally, the final prediction matrix only fits the original association from the mathematical layer and does not learn the deep representation among nodes.

The above two types of shallow methods have limited representation for complex biological data and lack the ability to learn essential features from sparsely known drug–disease associations (ratio of known associations to unknown associations was approximately 1 to 169 in our study) [19]. Series literatures found that deep learning methods are well suited for modeling complex biological data to support drug discovery [20,21,22]. In this study, we present CBPred, a novel method for predicting the potential drug–disease associations. First, we constructed a drug–disease heterogeneous network based on the similarities and known associations between nodes. Next, we proposed a novel two-way deep learning structure, a convolutional neural network (CNN), and bidirectional long short-term memory (BiLSTM)—named CBPred—for predicting and prioritizing candidate diseases of drugs. The original information and topological information among nodes were integrated using the CNN and BiLSTM to obtain deep representations and provide candidate diseases. An attention mechanism was introduced to improve the performance of our model because the contribution of different types of information to the drug–disease associations are different.

This novel method can deeply explore the original and topological representation of similarities between nodes, i.e., drugs and diseases, and known associations among two nodes. When we applied this method to various well-characterized drugs, CBPred recommended candidate diseases for treatment with the drugs with high accuracy. Case studies of five drugs, ciprofloxacin, ceftriaxone, ofloxacin, ampicillin, and levofloxacin, also demonstrated the ability of our method to recognize potential associations between drugs and diseases.

## 2. Materials and Methods

Our primary aim was to predict and prioritize novel association scores between drugs and diseases. We first constructed a drug–disease heterogeneous network via various connections among nodes, i.e., similarities and associations. To comprehensively consider original information and topological information of the drug–disease pair, we designed a novel prediction model based on the CNN module and BiLSTM module. Finally, we obtained association score between a drug *r_i_* and disease *d_j_*. A higher score indicated a greater likelihood that *r_i_* was involved in the disease process of *d_j_*.

### 2.1. Dataset

Drug–disease associations were obtained from a previous study [23], consisting of 763 drugs and 681 diseases. The drug–disease association data were originally extracted from the Unified Medical Language System [24]. There were 3051 known drug–disease associations. The chemical fingerprints for drug similarity calculations were extracted from PubChem [25]. Additionally, we used the method developed by Wang et al. [26] to construct directed acyclic graphs of the diseases using standard Medical Subject Headings disease terms.

### 2.2. Construction of a Drug–Disease Network

A two-layer heterogeneous drug–disease network, DrDisNet, was constructed based on the similarities and associations of drugs and diseases, which consisted of a drug network (DrNet) and disease network (DisNet) as well as the edge (i.e., association between drugs and diseases) among the two networks.

#### 2.2.1. Drug Network Construction

**Figure 1:**
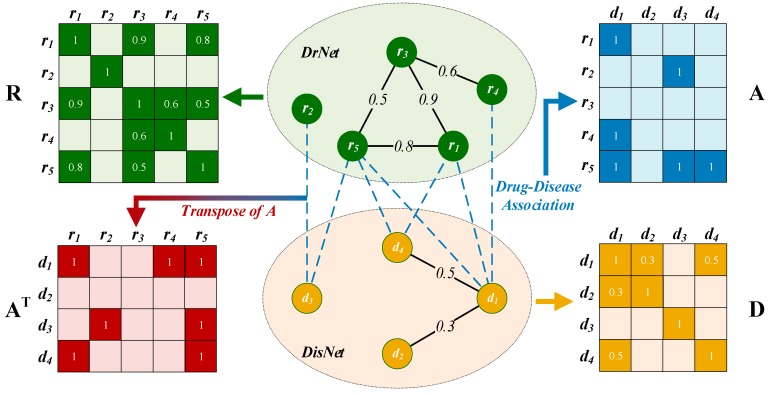
Construction of drug-disease heterogeneous network DrDisNet. ***R*** and ***D*** are the similarity matrix of drugs and diseases, respectively. ***A*** is the association matrix between drugs and diseases, while ***A*^T^** is the transpose of ***A***.

To measure the drug similarities for constructing the drug network (*DrNet*), we used the method developed by Liang et al. [1] to calculate the cosine similarity of the chemical substructure vector among the drugs. The chemical substructure vector of a drug is an 869-dimensional binary vector. The presence or absence of each chemical substructure of a drug is encoded as 1 or 0. When the drug similarity was greater than 0, we added an edge to connect the two drug nodes in *DrNet*; the weight of the edges reflected the similarity between the drugs (Figure 1). *DrNet* can be represented by matrix $R=\left[R_{ij}\right]\in R^{N_{r}\times N_{d}}$ where $N_{r}$ is the number of drugs and $R_{ij}$ is the similarity of drugs $r_{i}$ and $r_{j}$ in the range 0 to 1. An $R_{ij}$ closer to 1 indicates greater similarity between $r_{i}$ and $r_{j}$. $R_{ij}$ is calculated as follows:

(1) $$R_{ij}\;=\;\frac{c_{i}\cdot c_{j}}{||c_{i}||||c_{j}||}$$

where $c_{i}$ and $c_{j}$ are the chemical substructure vectors of $r_{i}$ and $r_{j}$, respectively, and ||·|| indicates the magnitude of vector.

#### 2.2.2. Disease Network Construction

Disease similarities play an important role in disease network construction. Wang et al. [26] used the MeSH disease term for each disease to calculate their respective semantic values. Next, semantic similarity was calculated from the semantic values of any two diseases. A larger number of common annotation terms among the two diseases indicated higher semantic similarity.

*DisNet* consisted of all pairs of diseases with similarity values greater than 0. The weight of any edge in the network was set to the similarity among the diseases to which the edge was connected. Matrix $D\in R^{N_{d}\times N_{d}}$ denotes DisNet where $D_{ij}$ is the similarity between diseases $d_{i}$ and $d_{j}$ and $N_{d}$ is the number of diseases. 

#### 2.2.3. Edges between DrNet and DisNet

We considered the known associations between drugs and diseases as the edges that connected the corresponding nodes in *DrNet* and *DisNet*. The edge set was represented as $A\in R^{N_{r}\times N_{d}}$, where each row represented a drug and each column represented a disease. $A_{ij}$ is 1 when drug $r_{i}$ has a known association with $d_{j}$, while it is 0 when an association is not observed between $r_{i}$ and $d_{j}$.

Finally, the heterogeneous drug–disease network *DrDisNet* was constructed by connecting *DrNet* and *DisNet* via known drug–disease associations (Figure 1). To concisely illustrate the subsequent methods, we assumed that $N_{r}$ = 5 and $N_{d}$ = 4.

### 2.3. Prediction Model Based on CNN and BiLSTM Module

**Figure 2:**
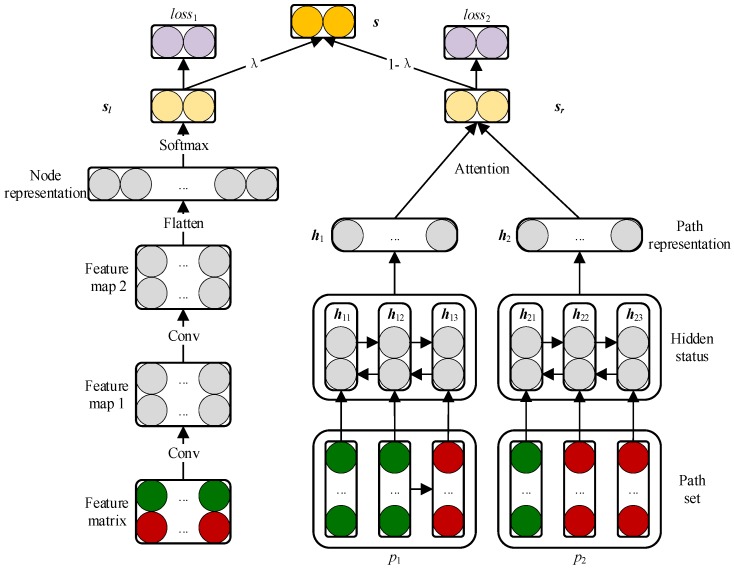
Construction of the framework based on the convolutional neural network and bidirectional long short-term memory for learning the original and path representations.

We propose a novel prediction model based on CNN and BiLSTM—named as CBPred—which is shown in Figure 2. The convolution module on the left part of CBPred was introduced to learn the association representation from the perspective of the original features of a node pair $\left(r_{i},\;d_{j}\right)$. Additionally, because the path from $r_{i}$ to $d_{j}$ also responds to the associated tendency between $r_{i}$ and $d_{j}$, a BiLSTM module on the right part was used to integrate topological information into the path representation.

#### 2.3.1. Embedding Layer

**Feature matrix of drug and disease for the CNN module.** Normally, if the similarity of a drug is more consistent with the association of a disease, the more likely it is that they are associated and vice versa. Therefore, we spliced up and down the similarities between the drug nodes and associations between drug and disease nodes, as shown on the left side of the feature matrix.

**Figure 3:**
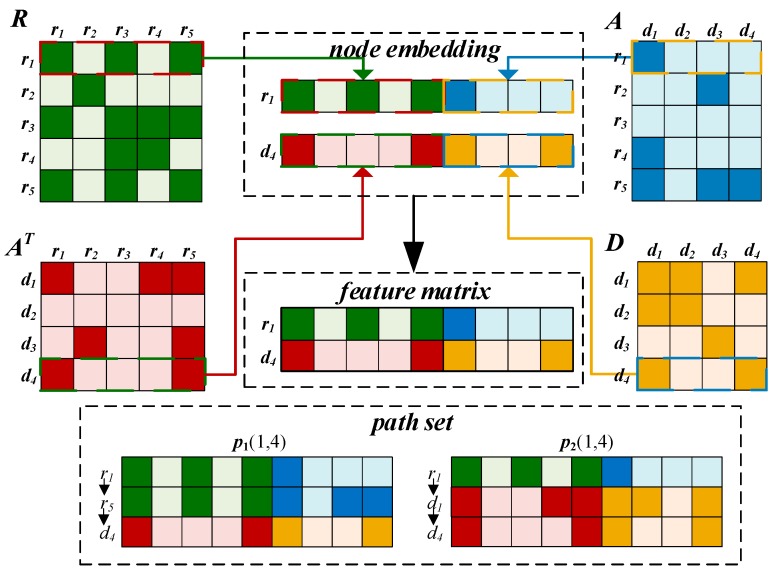
Integration process of drug and disease nodes to construct the feature matrix in the CNN module of our model and path set in the BiLSTM module of our model.

We use drug $r_{1}$ and disease $d_{4}$ as an example to illustrate the integration process (Figure 3). The first row of the drug similarity matrix R indicates the similarity to other drugs with $r_{1}$, and the fourth of the $A^{T}$ expresses the association drugs with $d_{4}$. Because $r_{1}$ is similar to $r_{4}$ and $r_{5}$, $r_{3}$ and $r_{5}$ are also both related to $d_{4}$. Thus, $r_{1}$ is likely to be involved in the disease process of $d_{4}$.

Similarly, if the relationship of $r_{1}$ and $d_{4}$ are more consistent with each disease, they will show a higher propensity for association. $r_{1}$ is associated with $d_{2\;}\;\mathrm{and}\;d_{3}$, while $d_{4}$ is similar to $d_{1}\;\mathrm{and}\;d_{3}$, and thus, $r_{1}$ may associate with $d_{4}$. Based on this information, we integrated the first row of A and the fourth row of D, as shown in the right part of the feature matrix. The final integration result is represented by the feature matrix $F\in R^{2\times \left(N_{r}+N_{d}\right)}$. Furthermore, the first and second rows of F are feature embedding of the drug and disease, respectively.

**Path sequence features for the BiLSTM module.** It is well known that if two drugs are very similar, they are likely involved in a similar disease process. For example, for the path, $r_{1}$–$r_{5}$–$d_{4}$, $r_{1}$ is similar to $r_{5},$ and $r_{5}$ is associated with $d_{4}$, indicating an association between $r_{1}$ and $d_{4}$. Based on similar logic, we can obtain the following path: Because $d_{3}$ is similar to $d_{4}$ and $r_{1}$ is associated with $d_{3}$, $d_{4}$ may be treated by $r_{1}$. Thus, there is a second path, $r_{1}$–$d_{3}$–$d_{4}$. Finally, we enumerate the path from the starting point $r_{s}$ to the end of $d_{t}$ in the network to obtain the path set $P_{\left(s,t\right)}\in R^{N_{\mathrm{path}}\;\times 1\;\times \;\left(N_{r}\;+{\;N}_{d}\right)}$, where $N_{path}$ is the number of paths between nodes $r_{s}$ and $d_{t}$, and the *i*-th path sequence in the $P_{\left(s,t\right)}$ defined as $p_{i}$. $P_{\left(1,4\right)}$ is inputted into the bidirectional LSTM module as the path feature of the pair $\left(r_{1},d_{4}\right)$ to learn the representation at the path level.

#### 2.3.2. Convolutional Module on the Left

**Figure 4:**
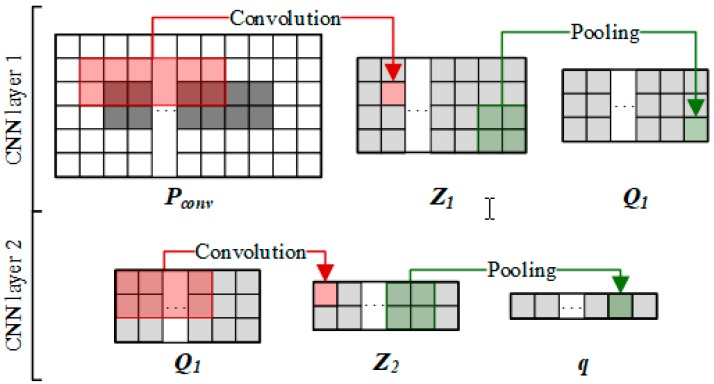
Learning process of the original representation of drug–disease pair by convolution and pooling on the left part.

The feature matrix  F is fed into the convolutional module to learn a latent original representation of node pair $\left(r_{1},d_{4}\right)$ (Figure 4). To capture the boundary information of F, we first pad F to obtain $P_{\mathrm{conv}}\in R^{\left(2\times p_{conv}+2)\times (2\times p_{conv}+N_{r}+N_{d}\right)}$, where $p_{conv}$ is the number of padding layers around F. For the first convolution layer, to apply the filter operators to the feature areas of $w_{h}\times w_{w}$, we set the size of filter as $\left(w_{h},w_{w}\right)$. 

Next, we can obtain the feature map $Z_{1}\in R^{(2\times p_{conv}-w_{h}+3)\times (2\times p_{conv}+N_{r}+N_{d}-w_{w}+1)\times N_{conv}}$ in this layer, where $N_{conv}$ is the number of filters. We used the subscript of the first element in the filter in $P_{conv}$ as the filter position. For example, $W_{conv}\left(i,\;j,k\right)$ indicates that the *k*th filter starts at the feature area at *i*th row and *j*th column in $P_{conv}$. The area and process of convolution are defined as follows:

(2) $$P_{conv}\left(i,\;j\right)\;=\;P_{conv}\left(i:i+w_{h}-1,\;j:j+\;w_{w}-\;1\right)$$

(3) $$Z_{1}i,j,k\;=g\left(P_{conv}\left(i,\;j\right)\;\times \;W_{conv}\left(i,\;j,k\right)+\;b_{conv}\left(k\right)\right)$$

(4) $$i\in \left[1,\;2\;+\;2\;\times \;p_{conv}\;-\;w_{h}\;+\;1\right],\;j\in \left[1,N_{r}+N_{d}+4-\;w_{w}\;+\;1\right],\;k\in \left[1,N_{conv}\right]$$

$Z_{1}(i,j,k)$ is the first convolution output in which the *k*th filter is sliding to the *i*th row and *j*th column of $P_{conv}$. g is a nonlinear activation function (rectified linear unit, ReLU), and ***b**_conv_* is a bias vector. To integrate features and reduce parameters, we use average pooling to compress the data in ***Z***_1_ in the pooling layer. The size of the pooling window is set to *a* × *b*, from which we obtain $Q_{1}\in R^{\frac{2\times p_{conv}-w_{h}+3}{a}\times \frac{2\times p_{conv}+N_{r}+N_{d}-w_{w}+1}{b}\times N_{conv}}$. We then use $Q_{1}$ as the input to the second convolution layer, and obtain a similar output $q\in R^{1\times \frac{2\times p_{conv}+N_{r}+N_{d}-w_{w}+1}{b}\times N_{conv}}$ through the second average pooling. q is then flattened to obtain an original representation of the node pair ($r_{1},d_{4}$), denoted as $v_{n}$:

(5) $$v_{n}\;=\;flatten\left(q\right)$$

#### 2.3.3. BiLSTM Module on the Right

**Figure 5:**
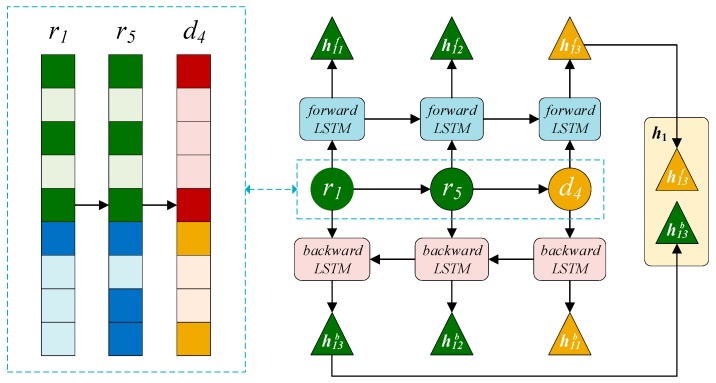
Learning process of the path representation in the BiLSTM module.

The LSTM module controls the information flow through the gate mechanism, while the BiLSTM module learns the context representation of the input sequence from a forward LSTM and reverse LSTM [27,28]. The previously obtained path set $P_{\left(1,4\right)}$ was fed into the BiLSTM module on the right part to learn the path representation of $r_{1}$ and $d_{4}$ (Figure 5). 

There are three gates, the forget gate $f_{ij}^{f}$, input gate $i_{ij}^{f}$, and output gate $o_{ij}^{f}$, in the forward LSTM unit which control how much information from path sequences should be forgotten, inputted, and outputted, respectively. The formulas for the three gates were defined as follows:

(6) $$\left[\begin{array}{c} f_{ij}^{f}\\ i_{ij}^{f}\\ o_{ij}^{f} \end{array}\right]\;=\;\left[\begin{array}{c} \sigma \\ \sigma \\ \sigma \end{array}\right]\left(W_{g}^{f}\left[h_{i\left(j-1\right)}^{f}⊕x_{ij}\right]+b_{g}^{f}\right)$$

where σ is the sigmoid activation function and ⊕ is the connection operator. The upper corner *f* indicates that this is a parameter of the forward LSTM unit; for example, $W_{g}^{f}$ and $b_{g}^{f}$ are the weight matrix and bias vector of the gate in the forward unit, respectively. $x_{ij}$ represents the embedding of the *j*th node of the *i*th path $p_{i}$ in the path set $P_{\left(1,4\right)}$.

Forward LSTM linearly integrates the candidate state ${\hat{c}}_{i\left(j\;-\;1\right)}^{f}$ of $x_{i\left(j\;-\;1\right)}$ with the candidate state ${\hat{c}}_{ij}^{f}$ of $x_{ij}$ and determines how much information in the ${\hat{c}}_{i\left(j\;-\;1\right)}^{f}$ should be retained by $f_{ij}^{f}$ and how much information in the ${\hat{c}}_{ij}^{f}$ are accepted by $i_{ij}^{f}$. Thus, obtaining the state $c_{ij}^{f}$ of the sequence consisting of the 1st to *j*th nodes in the $p_{i}$:

(7) $$c_{ij}^{f}\;=\;f_{ij}^{f}⨀{\hat{c}}_{i\left(j-1\right)}^{f}+\;i_{ij}^{f}⨀{\hat{c}}_{ij}^{f}$$

where ⨀ is the element-wise product operator. The candidate state ${\hat{c}}_{ij}^{f}$ of $x_{ij}$ is obtained by comprehensively considering the information from the previous node and $x_{ij}$, defined as follows:

(8) $${\hat{c}}_{ij}^{f}\;=\tanh\left(W_{c}^{f}\left(h_{i\left(j-1\right)}^{f}⊕x_{ij}\right)\;+\;b_{c}^{f}\right)$$

where $W_{c}^{f}$ and $b_{c}^{f}$ are the weight matrix and bias vector of the candidate state, respectively. Finally, how much information in $c_{ij}^{f}$ is adjusted by $o_{ij}^{f}$ as the hidden state $h_{ij}^{f}$ output is expressed as follows:

(9) $$h_{ij}^{f}\;=\;\tan h\left(o_{ij}^{f}⨀c_{ij}^{f}\right)$$

where $h_{ij}^{f}$ is a forward path representation of the 1st to *j*th nodes in $p_{i}$. We take the hidden state $h_{il}^{f}$ of the last node as the representation of $p_{i}$, where l is the length of $p_{i}$. The inverted sequence $p_{i}^{b}$ of $p_{i}$ is then inputted into a structurally similar backward LSTM module to obtain a backward representation $h_{il}^{b}$ of $p_{i}^{b}$. The upper corner *b* indicates that this is a parameter of the backward LSTM module. Thus, the path representation of the *i*th path in the bidirectional LSTM module is given by the following formula:

(10) $$h_{i}\;=\;h_{il}^{f}⊕h_{il}^{b}.$$

#### 2.3.4. Attention Mechanism at Path Level

From the perspective of $P_{\left(1,4\right)}$, not all paths equally contributed to the association prediction of $r_{1}$ and $d_{4}$. An attention mechanism at the path level was introduced to extract paths important in the association between the drug and disease [29]. This yields:

(11) $$u_{i}\;=\;\tanh\left(W_{p}h_{i}+b_{p}\right)$$

(12) $$\alpha_{i}\;=\;\frac{exp\left(u_{i}u_{p}^{T}\right)}{\sum_{j}exp\left(u_{j}u_{p}^{T}\right)}$$

(13) $$v_{p}\;=\;\underset{i}{\sum }\alpha_{i}h_{i}$$

where $u_{i}$ is a hidden representation of $h_{i}$. The path level context vector $u_{p}$ attempts to generalize the path strongly contributing to the association between r1 and d4 from $P_{\left(1,4\right)}$, while $u_{p}^{T}$ is the transpose of $u_{p}$. Next, we measured the importance of $p_{i}$ in $P_{\left(1,4\right)}$ by comparing the similarity between $u_{i}$ and $u_{p}$, and obtained the attention weight $\alpha_{i}$ through the softmax function. $v_{p}$ is a path vector, which is a weighted sum of all information from path set $P_{\left(1,4\right)}$ based on the attention weights and path representations.

#### 2.3.5. Combined Strategy

The original representation $v_{n}$ and path representation $v_{p}$ are both high-level representations of $r_{1}$ and $d_{4}$ and can be used as features for association classification. Thus, we projected the two representations $v_{n}$ and $v_{p}$ into the association distribution of *C* classes via the SoftMax layer while choosing the cross-entropy loss to evaluate the error between the known association distribution and prediction distribution:

(14) $$s_{n}\;=\;softmax\left(W_{n}v_{n}\;+\;b_{n}\right)$$

(15) $$loss_{n}\;=\;-\underset{t\in T}{\sum }\overset{C}{\underset{c\;=\;0}{\sum }}p_{c}^{g}\left(t\right)\times \log(s_{n}\left(t\right))$$

(16) $$s_{p}\;=\;softmax\left(W_{p}v_{p}+b_{p}\right)$$

(17) $$loss_{p}\;=\;-\;\underset{t\in T}{\sum }\overset{C}{\underset{c\;=\;0}{\sum }}p_{c}^{g}\left(t\right)\times \log(s_{p}\left(t\right))$$

where t is the node pair in the training set T, $p_{c}^{g}\left(t\right)$ is the one hot embedding of t, and $s_{n}\left(t\right)$ and $s_{p}\left(t\right)$ are the predicted scores of t from the CNN and BiLSTM modules, respectively. We designed a combined strategy for the model to make full use of the original representation $v_{n}$ and path representation $v_{p}$. We used the Adam optimization algorithm to optimize the objective function [30]. Let λ be a hyperparameter to control the contribution of the original representations and path representations of the node pairs for the final predicted score.

(18) $$s\;=\;\lambda s_{n}+\left(1-\lambda \right)s_{p}$$

## 3. Experimental Evaluation and Discussion

### 3.1. Evaluation Metrics

We performed 5 fold cross-validation 20 times to evaluate the performance of our prediction method and the corresponding results were averaged [31,32]. First, known associated drug–disease pairs were divided randomly into five subsets and treated as positive samples. The remaining pairs were considered negative samples. Because the number of positive samples was much smaller than the number of negative samples in our dataset (approximately 1 to 169), we sampled a matching number of non-associating pairs randomly and divided them into five subsets to reduce the impact of class imbalance in predicting the results. Particularly, in each fold cross-validation, we used four positive and negative subsets as the training set for model training and the remaining positive samples as the testing set for performance evaluation. Finally, a higher rank for the positive samples indicated better the prediction performance of the method.

A disease with a score higher than the threshold *θ* indicates that it is identified as a positive sample and vice versa. Thus, the *TPR*s (true-positive rates) and *FPR*s (false-positive rates) under various *θ* can be calculated as follows:

(19) $$TPR\;=\frac{TP}{TP\;+\;FN},\;FPR\;=\frac{FP}{TN\;+\;FP}$$

where *TP* (true-positive) and *TN* (true-negative) are the number of positive and negative samples which were correctly identified, while *FN* (false-negative) and *FP* (false-positive) are the number of positive and negative samples which were misidentified [33]. The receiver operating characteristic (ROC) curve can be drawn according to the *TPR* and *FPR* under each *θ* [34]. 

A ROC curve was constructed for each drug, and the area under the ROC curve (AUC) was used to evaluate the predictive performance of the method for the specific drug [35,36]. The average AUC of all drugs is considered as the comprehensive performance of the prediction model.

However, in most cases of class imbalance, the precision–recall (P–R) curves are more informative than the ROC curve [37]. Precision is the proportion of true-positive samples in all identified positives and recall is the ratio of true-positives among the samples with known associations [38]. Therefore, we used the P–R curve as another measurement to evaluate the performance of each method. The area under the P–R curve (AUPR) is another evaluation metric that focuses on true-positive samples [39]. The precision rates and recall rates can be defined as follows:

(20) $$Precision\;=\frac{TP}{TP\;+\;FP},\;Recall\;\;=\frac{TP}{TP\;+\;FN}.$$

Additionally, biologists typically select the top part of the predictive result for further validation in wet-lab experiments. Thus, the recall rates of the top *k* candidate drug-related diseases are more important because they reveal the number of successfully identified positive samples. We calculated the recall rates of the top *k* candidate to demonstrate the performance of each method on the top rankings of the predictive result.

### 3.2. Comparison with Other Methods

To evaluate the performance of CBPred, we compared this method with a series of state-of-the-art methods for predicting associations between drugs and diseases, including MBiRW [15], LRSSL [1], SCMFDD [18], and HGBI [16].

The hyperparameter of CBPred, λ, was selected from {0.1, 0.2, …, 0.9}. Since CBPred yielded better performances for both λ = 0.1 and 0.2, we chose 0.12 as the final value of λ after fine tuning. The learning rate was set as 0.001. For the first convolutional layer, we set the kernel size = (3, 5), out channel = 16, and pooling size = 2. For the second convolutional layer, kernel size = (3, 11), out channel = 32, and pooling size = 2. For fair comparison, the parameters in other methods were adjusted according to the authors’ suggestions (i.e., *α* = 0.3, *c* = −11, *d* = log(9999), *l* = *r* = 2 for MBiRW, *μ* = *λ* = 0.01, *γ* = 2, *k* = 10 for LRSSL, *k* = 45%, *μ* = 1, *λ* = 4 for SCMFDD, and *α* = 0.4 for HGBI).

**Figure 6:**
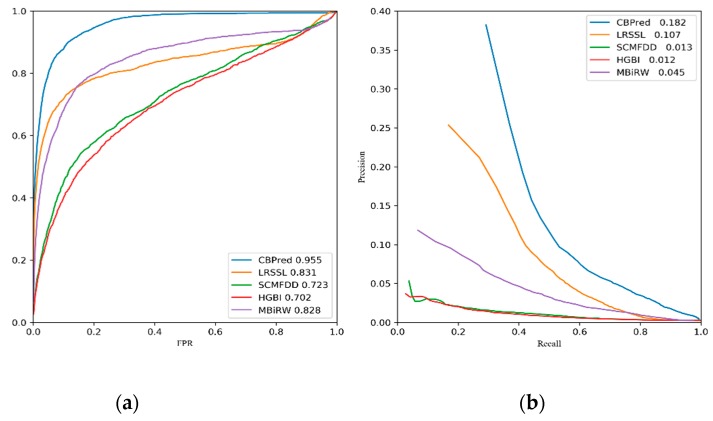
Two type of curves of CBPred and other methods for predicting performance evaluation. (**a**) Receiver operating feature characteristic (ROC) curves; (**b**) precision–recall (P–R) curves.

As shown in Figure 6a, CBPred showed the best performance for 763 drugs (AUC = 0.955). Specifically, CBPred showed a 25.3% higher AUC than HGBI, 23.2% higher AUC than SCMFDD, 12.7% higher AUC than MBiRW, and 12.4% higher AUC than LRSSL. We also show the predictive results of 15 well-characterized drugs in Table 1; CBPred achieved the best performance for 12 drugs. Both CBPred and LRSSL not only consider the nodes’ attributes based on node similarities, but also extract topological information of drug–disease heterogeneous networks. Thus, compared to other methods, CBPred and LRSSL achieved the best and second-best performances. Luo et al. constructed a random walk with a restart-based model, MBiRW, for predicting associations between drugs and diseases. It focuses on the topological information of the networks, while node attributes are ignored. Additionally, because the restart probability is difficult to determine, which may result in insufficient global topological information or excessive noise, the performance of MBiRW was worse than the second method, LRSSL. Zhang et al. applied a matrix factorization-based model, SCMFDD, for predicting novel associations, which relies on the adjacency matrices of the heterogeneous network. However, reducing the dimension of the feature vectors may lead to loss of the potential information. Thus, the performance of SCMFDD was worse than that of MBiRW but better than that of HGBI. Comprehensively, HGBI showed lower performance than the other methods because it was too dependent on the similarity of drugs and diseases.

The precision–recall curves of each method are demonstrated in Figure 6b. The average AUPR of CBPred was greater than those of all the other methods (AUPR = 0.182). Our method, CBPred, achieved a 17.0%, 16.9%, 13.7%, and 7.5% higher AUPR than HGBI, SCMFDD, MBiRW, and LRSSL, respectively. As shown in Table 2, CBPred showed the best performance for 12 of the 15 well-characterized drugs.

A Wilcoxon test to evaluate the prediction results of 763 drugs revealed that CBPred significantly outperformed the other methods [40,41,42]. These results were observed using a *p*-value threshold of 0.05, with CBPred showing better performance in terms of both AUCs and AUPRs (Table 3).

**Figure 7:**
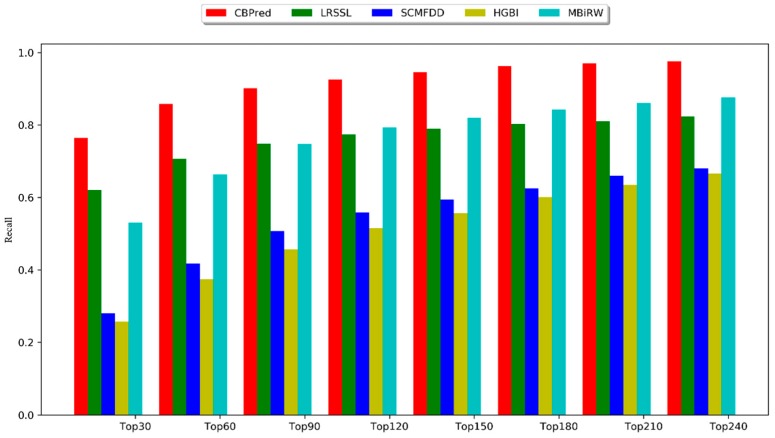
Top *k* recall rate of CBPred and other methods.

Among the top *k*-ranked drugs, a higher recall rate indicated that drug-associated diseases were correctly identified. Our method, CBPred, consistently outperformed the other methods under different *k* values, as shown in Figure 7, and ranked 76.38% for the top 30 drugs, 85.78% for the top 60, and 92.54% for the top 120. Zhang’s method, SCMFDD, showed very similar results to Wang’s method, HGBI, for most of the recall rates, with the former ranked 27.97%, 41.75%, and 55.82% for the top 30, 60, and 120 drugs, respectively, while the latter ranked 25.70%, 37.39%, and 51.57%. The recall of LRSSL was higher than that of MBiRW before the top 120, after which it was surpassed. This may be because the *k*-nearest neighbors algorithm is utilized in the process of LRSSL, which may make the prediction effect too dependent on neighboring node information, causing difficulties in predicting isolated nodes. Luo’s method, MBiRW, captured the global information for the drug–disease network and local topology of the node through random walk with restart algorithm, which showed better results than LRSSL.

In addition, to confirm the performance of CBPred from another perspective, we constructed a new drug–disease network where the disease similarities are calculated using disease ontology and disease-related genes according to Cheng’s method [43]. The ROC and P–R curves of CBPred and other methods are shown in Supplementary Materials Figure S1. Our method, CBPred, still achieved the best performance under the new drug–disease network, which also illustrated that CBPred was effective when the disease ontology and disease-related genes were taken into account.

### 3.3. Case Studies of Five Drugs

To demonstrate the ability of CBPred to discover novel drug–disease associations, we conducted case studies of ciprofloxacin, ceftriaxone, ofloxacin, ampicillin, and levofloxacin and then analyzed their top ten candidate diseases (Table 4).

The impacts of chemicals (i.e., drugs) on human health are presented in the Comparative Toxicogenomics Database (CTD). This information was manually collected and verified from published works. DrugBank records various attributes of the drug itself, such as associations with diseases. As shown in Table 3, 12 candidates are supported by direct evidence in CTD, and 9 candidates are involved according to DrugBank. These records indicate that these candidate diseases are treated with the corresponding drugs.

Clinical Trials is a database of clinical trials conducted worldwide and provides access to various ongoing and completed experimental information, with detailed patient descriptions and experimental dosing regimens and treatment outcomes. We selected only records with a status of “Completed” as our support material. The clinical trial results showed that our drug has a therapeutic relationship with the candidate disease. PubChem is a public database containing information on chemicals and their biological activities and is supported by the National Institutes of Health. Fifteen candidates were included from Clinical Trials and 11 candidates were included by PubChem. This demonstrated that the candidates are supported by clinical trials.

In addition to the manually verified drug–disease associations, the CTD database also contains inferred associations from literature that are temporarily unconfirmed. Four candidates were included by the inferred part of CTD, which shows that they are likely to have associations. Direct or indirect descriptions of all disease candidates for five drugs were found, revealing that CBPred can identify drug–disease association candidates with high reliability and accuracy.

### 3.4. Prediction of Novel Drug–Disease Associations

After evaluating CBPred’s prediction performance through five-fold cross-validation, case studies, and Wilcoxon test, we applied CBPred to all drugs. All known drug–disease associations were considered as the training set to train CBPred’s prediction model. Many high-confidence candidate diseases of drugs were obtained via CBPred and are listed in Supplementary Materials Table S1.

## 4. Conclusions

A novel method based on a CNN and BiLSTM—CBPred—was developed for predicting potential disease indications for drugs. The CNN module of the CBPred captures complex and non-linear relationships among drug similarities, disease similarities, and drug–disease associations about a drug–disease pair. The path information was deeply integrated using the BiLSTM module of this method. We also established an attention mechanism at the path level to discriminate the different contributions of the path, which enhanced the prediction performance of CBPred. The experimental results revealed that CBPred outperformed other state-of-the-art methods in terms of both AUCs and AUPRs. Case studies of five drugs confirmed the ability of CBPred to discover potential disease indications for drugs. Our method, CBPred, is a prioritization tool that identifies reliable candidate drug–disease associations for subsequent biological validation in wet-lab experiments.

## Figures and Tables

**Table 1:** Prediction results of CBPred and four other methods for 15 drugs in terms of the area under the receiver operating characteristic curve (AUC).

Disease Name	AUC				
	CBPred	LRSSL	SCMFDD	HGBI	MBiRW
Ave AUC on 763 drugs	**0.955**	0.831	0.723	0.702	0.828
ampicillin	**0.909**	0.885	0.861	0.786	0.906
cefepime	**0.953**	0.932	0.898	0.910	0.872
cefotaxime	0.906	0.902	0.911	0.870	**0.967**
cefotetan	0.889	0.892	0.897	**0.908**	0.866
cefoxitin	**0.913**	0.911	0.899	0.909	0.907
ceftazidime	**0.940**	0.925	0.939	0.924	0.916
ceftizoxime	**0.902**	0.894	0.841	0.823	0.854
ceftriaxone	0.863	**0.925**	0.808	0.779	0.851
ciprofloxacin	**0.917**	0.893	0.810	0.790	0.844
doxorubicin	**0.921**	0.749	0.361	0.486	0.918
erythromycin	**0.859**	0.817	0.769	0.734	0.857
itraconazole	**0.942**	0.543	0.701	0.560	0.897
levofloxacin	**0.910**	0.852	0.824	0.819	0.867
moxifloxacin	**0.909**	0.792	0.841	0.849	0.826
ofloxacin	**0.899**	0.884	0.851	0.845	0.896

The bold values indicate the higher AUCs.

**Table 2:** Prediction results of CBPred and four other contrast methods for 15 drugs in terms of the area under the precision–recall curve (AUPR).

Disease Name	AUPR				
	CBPred	LRSSL	SCMFDD	HGBI	MBiRW
Ave AUPR on 763 drugs	**0.182**	0.107	0.013	0.012	0.045
ampicillin	**0.249**	0.220	0.059	0.089	0.058
cefepime	0.258	**0.562**	0.101	0.137	0.279
cefotaxime	**0.276**	0.273	0.072	0.098	0.266
cefotetan	0.177	**0.724**	0.093	0.131	0.152
cefoxitin	**0.227**	0.136	0.051	0.081	0.186
ceftazidime	**0.201**	0.187	0.132	0.164	0.119
ceftizoxime	**0.328**	0.168	0.125	0.174	0.153
ceftriaxone	**0.269**	0.138	0.081	0.101	0.123
ciprofloxacin	**0.471**	0.256	0.061	0.074	0.071
doxorubicin	**0.164**	0.159	0.006	0.007	0.075
erythromycin	**0.194**	0.034	0.013	0.013	0.052
itraconazole	**0.334**	0.057	0.008	0.006	0.097
levofloxacin	0.263	**0.512**	0.086	0.111	0.177
moxifloxacin	**0.301**	0.158	0.095	0.126	0.098
ofloxacin	**0.221**	0.214	0.114	0.158	0.095

The bold values indicate the higher AUPRs.

**Table 3:** Results of Wilcoxon test on CBPred and four other contrast methods for 763 drugs.

*p*-Value between CBPred and Another Method	LRSSL	SCMFDD	HGBI	MBiRW
*p*-value of ROC curve	3.577 × 10^−13^	1.218 × 10^−75^	1.460 × 10^−80^	3.724 × 10^−32^
*p*-value of P–R curve	2.591 × 10^−15^	1.122 × 10^−76^	6.075 × 10^−80^	4.577 × 10^−38^

**Table 4:** The top 10 candidates of 5 popular drugs supported by databases. The associations involved in the table are all inferred by the literature in the comparative toxicogenomic database or included by databases.

	Rank	Disease Name	Description	Rank	Disease Name	Description
Ciprofloxacin	1	Conjunctivitis, Bacterial	ClinicalTrials	6	Campylobacter Infections	Drugbank
	2	Chlamydia Infections	CTD	7	Neurocysticercosis	Drugbank
	3	Thrombocytopenic, Idiopathic	Drugbank	8	Respiration Disorders	ClinicalTrials
	4	Acanthamoeba Keratitis	Drugbank	9	Anthrax	CTD
	5	Scalp Dermatoses	PubChem	10	Skin Diseases	CTD
Ceftriaxone	1	Panic Disorder	Drugbank	6	Bacteroides Infections	PubChem
	2	Respiration Disorders	ClinicalTrials	7	Bone Diseases, Infectious	ClinicalTrials
	3	Respiratory Distress Syndrome, Adult	ClinicalTrials	8	Multiple Myeloma	Drugbank
	4	Rickettsia Infections	PubChem	9	Rectal Neoplasms	inferred candidate by 2 literature
	5	Respiratory Distress Syndrome, Newborn	ClinicalTrials	10	Maxillary Sinusitis	Drugbank
Ofloxacin	1	Trichuriasis	inferred candidate by 1 study	6	Pulmonary Valve Stenosis	PubChem
	2	Corneal Ulcer	PubChem	7	Schizophrenia	CTD
	3	Nausea	CTD	8	Peritonitis	CTD
	4	Rectal Neoplasms	ClinicalTrials	9	Mouth Diseases	CTD
	5	Epididymitis	Drugbank	10	Proteus Infections	CTD
Ampicillin	1	Keratosis	inferred candidate by 1 literature	6	Pneumonia, Bacterial	CTD, ClinicalTrials
	2	Bacterial Infections	CTD	7	Toothache	ClinicalTrials
	3	Respiratory Syncytial Virus Infections	inferred candidate by 1 study	8	Respiratory Tract Fistula	PubChem
	4	Respiratory Tract Diseases	ClinicalTrials	9	Mouth Diseases	ClinicalTrials
	5	Burns	CTD	10	Sarcoma, Ewings	PubChem
Levofloxacin	1	Pneumonia, Mycoplasma	ClinicalTrials	6	Respiratory Syncytial Virus Infections	CTD
	2	Rhinitis	PubChem	7	Soft Tissue Infections	Drugbank
	3	Bacteroides Infections	PubChem	8	Respiratory Tract Fistula	PubChem
	4	Tuberculosis, Pulmonary	ClinicalTrials	9	Listeriosis	PubChem
	5	Respiratory Tract Diseases	ClinicalTrials	10	Mouth Diseases	ClinicalTrials

## Supplementary Materials

The following are available online at https://www.mdpi.com/2073-4409/8/7/705/s1. Table S1: The top 10 potential candidates for 763 drugs. Figure S1: Two type of curves of CBPred and other methods under a new drug–disease network.
