# Supplementary material for: Convolutional Neural Network and Bidirectional Long Short-Term Memory-Based Method for Predicting Drug–Disease Associations
Source: Cells. 2019 Jul 11;8(7):705. doi: 10.3390/cells8070705 (PMC6679344; doi:10.3390/cells8070705)
Supplement: Supplementary file 1 [file cells-08-00705-s001.zip › FigureS1.pdf]

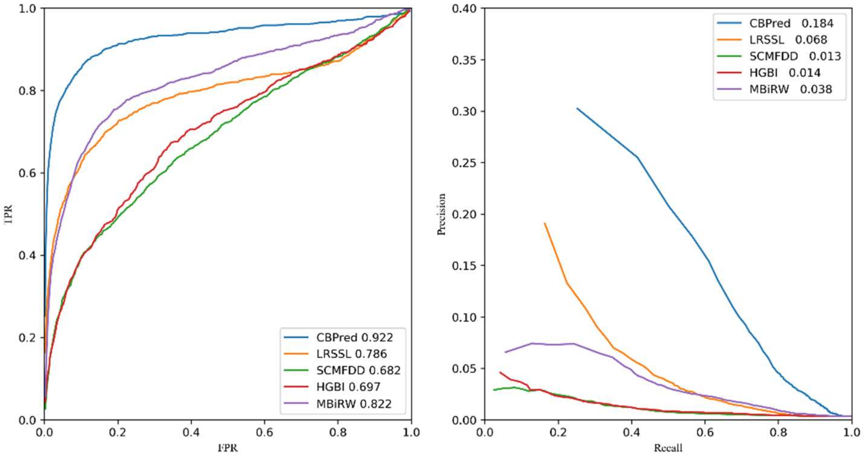

(**a**) (**b**)

Figure S1. Two type of curves of CBPred and other methods under the new drug-disease network. **(a)** Receiver operating feature characteristic (ROC) curves; **(b)** Precision–recall (P–R) curves.
